# Loss of Sex and Age Driven Differences in the Gut Microbiome Characterize Arthritis-Susceptible *0401 Mice but Not Arthritis-Resistant *0402 Mice

**DOI:** 10.1371/journal.pone.0036095

**Published:** 2012-04-24

**Authors:** Andres Gomez, David Luckey, Carl J. Yeoman, Eric V. Marietta, Margret E. Berg Miller, Joseph A. Murray, Bryan A. White, Veena Taneja

**Affiliations:** 1 Institute for Genomic Biology, University of Illinois, Urbana, Illinois, United States of America; 2 Department of Immunology, Mayo Clinic, Rochester, Minnesota, United States of America; 3 Department of Gasteroenterology, Mayo Clinic, Rochester, Minnesota, United States of America; 4 Department of Rheumatology, Mayo Clinic, Rochester, Minnesota, United States of America; 5 Department of Animal Sciences, University of Illinois, Urbana, Illinois, United States of America; Baylor College of Medicine, United States of America

## Abstract

**Background:**

HLA-DRB1*0401 is associated with susceptibility, while HLA-DRB1*0402 is associated with resistance to developing rheumatoid arthritis (RA) and collagen-induced arthritis in humans and transgenic mice respectively. The influence of gut-joint axis has been suggested in RA, though not yet proven.

**Methodology/Principal Findings:**

We have used HLA transgenic mice carrying arthritis susceptible and -resistant HLA-DR genes to explore if genetic factors and their interaction with gut flora gut can be used to predict susceptibility to develop arthritis. Pyrosequencing of the 16S rRNA gene from the fecal microbiomes of DRB1*0401 and DRB1*0402 transgenic mice revealed that the guts of *0401 mice is dominated by a Clostridium-like bacterium, whereas the guts of *0402 mice are enriched for members of the *Porphyromonadaceae* family and *Bifidobacteria*. DRB1*0402 mice harbor a dynamic sex and age-influenced gut microbiome while DRB1*0401 mice did not show age and sex differences in gut microbiome even though they had altered gut permeability. Cytokine transcripts, measured by rtPCR, in jejuna showed differential TH17 regulatory network gene transcripts in *0401 and *0402 mice.

**Conclusions/Significance:**

We have demonstrated for the first time that HLA genes in association with the gut microbiome may determine the immune environment and that the gut microbiome might be a potential biomarker as well as contributor for susceptibility to arthritis. Identification of pathogenic commensal bacteria would provide new understanding of disease pathogenesis, thereby leading to novel approaches for therapy.

## Introduction

Rheumatoid arthritis (RA) is a chronic inflammatory disease that is characterized by synovial inflammation and erosion of bone and cartilage leading to the destruction of joints. Although the etiology of RA is unknown, both genetic and environmental factors contribute to the susceptibility to developing arthritis [1]. Among the known genetic factors, strong associations are observed between RA and the presence of certain HLA-DR alleles that share the 3^rd^ hypervariable region with DRB1*0401 gene, known as the ‘shared epitope’ hypothesis. In contrast, DRB1*0402 confers resistance to the development of arthritis. Some evidence points to an infectious etiology for RA, such the presence of certain oral and gut commensal bacterial antigens in synovial fluids of patients [1], [2], [3]. Migration of gut commensals or their products to peripheral organs may be facilitated by loss of intestinal integrity, resulting in mucosal or systemic immune stimulation. Recent studies have shown that specific intestinal commensals or their specific molecular patterns may modulate the integrity of the intestinal mucosal barrier by inducing the expression of pro or anti-inflammatory cytokines [4], [5]. Thus, alterations of a normal gut microbiome can affect mucosal immunity and have an extended effect on non-intestinal diseases like diabetes and RA [6]. For instance, previous analysis of the fecal microbiome of patients with RA revealed significantly fewer *Bifidobacterium* and bacteria of the *Bacteroides-Porphyromonas-Prevotella* group, *B. fragilis* subgroup, and the *Eubacterium rectale–Clostridium coccoides* group than the fecal microbiota of patients with non-inflammatory fibromyalgia [7]. Because these bacterial species are known to belong to common taxa in the human fecal microbiome [8], [9], [10], their low levels in RA patients might suggest an altered gut microbiome. Further, specific gut commensals such as *Bifidobacterium infantis*, can induce an anti-inflammatory response in the intestinal mucosal and peripheral immune systems by suppressing T-cell proliferation and production of IL-10 and Th2 cytokines, and by inhibiting nuclear factor kappa B (NF-κB) activation [11], [12], [13]. Although dendritic cells (DCs), directly in contact with intestinal lumen contents, can instruct naive CD4+ T-cells to differentiate into Th1, Th17, Th2 or T-regulatory cells, a unique gastro-intestinal environment may favor the proliferation of the latter, a process possibly dependent on the presence of specific gut commensal bacteria thus setting up the basis for immunotolerance [14], [15], [16].

We have generated two lines of HLA transgenic mice carrying the RA-susceptible DRB1*0401 and RA-resistant DRB1*0402 genes that lacked all four classical murine chains, Aα, Aβ, Eα, Eβ. Because human class II molecules shape the T-cell repertoire in these humanized mice, they show the same HLA restrictions in an immune response as humans [17], [18], [19]. Upon immunization with type II collagen (CII), *0401 mice develop collagen-induced arthritis (CIA), while *0402 mice do not. The most remarkable features of CIA in *0401 mice that is not observed in any other model is a sex-bias in the onset of arthritis with a ratio of 3 Females: 1 Male, production of rheumatoid factor (RF) and anti-cyclic citrullinated peptide antibodies (ACPAs), diagnostic markers for RA patients [18], [20]. Host MHC genes affect the microbial composition of the gut [21], [22]. However, the interactions between host genetic factors like MHC and their gut microbiota, and their impact on the development of RA are difficult to study in humans due to several factors that include: high HLA polymorphism, diet and the fact that the disease is well established at the time of diagnosis. Few studies describing microbiome of RA patients have not done tag sequencing and also not analyzed the data according to sex and age. Thus, HLA transgenic mice described here provide a useful tool to understand the role of gut microbiota in the pathogenesis of RA. Herein, we show that mice with the RA-susceptible DRB1*0401 gene harbor altered patterns of gut microbiome characterized by an abundance and/or lack of specific commensals as compared to mice with the RA-resistant DRB1*0402 gene whose gut microbiomes are shaped by age and sex. A differential expression of Th17 regulating gene transcripts, a compromised gut permeability in *0401 mice and observed dysbiosis in *0401 mice may in combination or independently contribute to susceptibility to arthritis.

## Results

### Arthritis-susceptible DRB1*0401 and –resistant DRB1*0402 mice differ in their gut microbiomes

The gut microbiome plays a crucial role in the homeostasis of the immune system and is also linked to gut permeability. We tested if an arthritis-susceptible genotype may be associated with the presence or absence of specific gut bacteria by sequencing the microbiome community structure in fecal samples of 87 mice (n = 41 for *0401 and n = 45 for *0402 mice) using Roche 454 GS-FLX Titanium Pyrosequencing technology. This included both male and female mice of various ages for both strains. After processing, 568,571 high quality sequences were used for further analysis (sequence lengths ranged from 417 to 534 bp with a 506 median sequence length). A total of 5,267 operational taxonomic units (OTUs) clustered at 97% sequence similarity were used for microbiome analysis (1,953 to 60,915 reads per sample).

**Figure 1:**
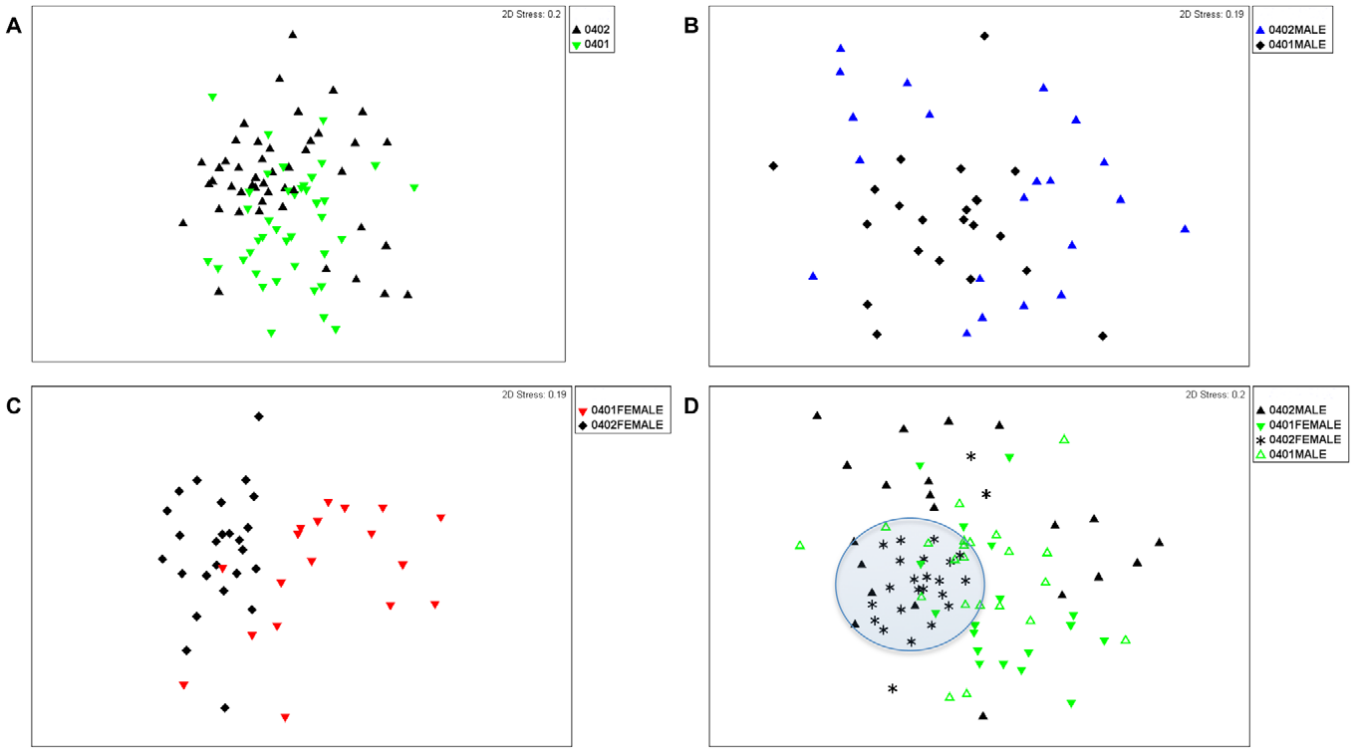
Multidimensional Scalinge analysis of the fecal microbiomes of arthritis-susceptible *0401 and –resistant *0402 mice. 16S-rDNA bacterial community structure differences can be visualized with each symbol representing data from a single mouse fecal sample. (**a**) 16S-rDNA bacterial community structures between *0401 (n = 41) and *0402 (n = 45) mice do not differ significantly (ANOSIM R = 0.14). (**b**) *0401 (n = 22) and *0402 (n = 21) males do not show significant differences in fecal microbiome structure (ANOSIM R = 0.14), while (**c**) fecal microbiomes of *0401 (n = 19) and *0402 (n = 24) females differ significantly (ANOSIM R = 0.436). (**d**) Shaded area shows that the fecal microbiomes of *0402 females are compact and may be driving differences between both genotypes.

**Figure 2:**
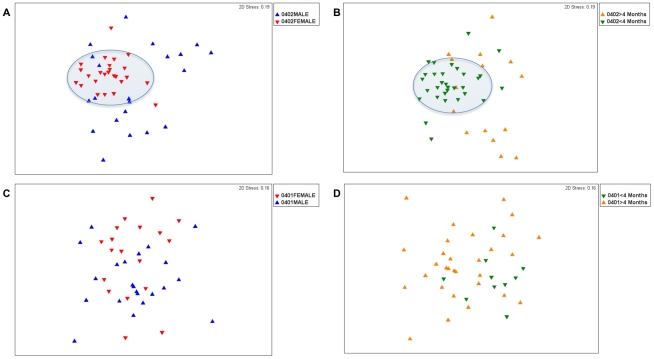
Sex and Age based Multidimensional Scalinge analysis of fecal microbiomes. (**a**) *0402 mice show significantly different fecal microbiome structure according to sex (ANOSIM R = 0.302) and (**b**) age (ANOSIM R = 0.423). (**c**) *0401 mice lost sex and (**d**) age-based differences in fecal microbiome (ANOSIM R = 0.052 and R = 0.043 respectively). Shaded areas show compact microbiome structures.

**Figure 3:**
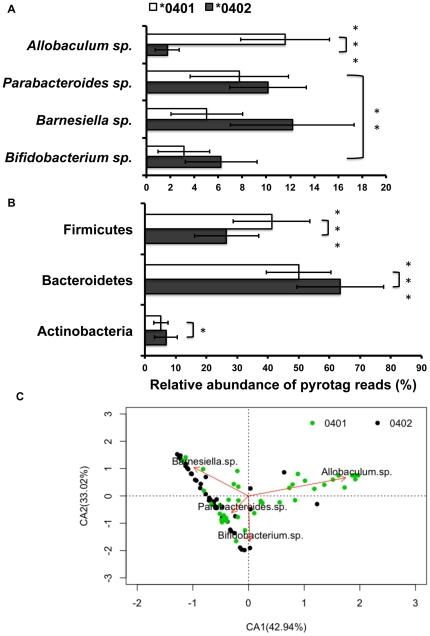
Relative abundance of OTUs in the fecal microbiomes of *0401 and *0402 mice. (**a**) *Allobaculum sp*. is is the most abundant OTU in the *0401 mice, while *Barnesiella* sp. occurs with highest frequency in *0402 mice (P<0.05). (**b**) Similar taxa distributions are observed at phyla level with Bacteroidetes: Firmicutes ratios more even in *0401 (n = 41) compared to *0402 (n = 45) mice. Data are presented as mean ± S.E., *P<0.05, **P<0.01, ***P<0.001. (**c**) Correspondence analysis plot shows the degree of correlation between specific OTUs and mice genotype. The red vectors point to the center of gravity of the samples where each OTU mostly occurs. The distance between the tip of the vector and the samples (dots) give an indication of the probability of OTU content in each sample. Green and black dots represent *0401 and *0402 fecal samples respectively. Percentages in parentheses in the CA plots describe the amount of variation explained by each axis.

Non-metric multidimensional scaling (NMDS) and analysis of similarities (ANOSIM) suggest that *0401(n = 41) and *0402 (n = 45) mice display only minor differences in their fecal microbiome profiles (ANOSIM R-statistic = 0.14, P = 0.001) (Figure 1 a). There were no significant differences in bacterial richness between the 2 strains (P>0.1). However, the fecal bacterial species in *0401 mice were slightly more evenly distributed than those in *0402 mice (Shannon evenness index, P = 0.04). Both NMDS and ANOSIM analysis of males and females from each strain showed that sex was a confounding factor and that males were masking the differences between the fecal bacterial profiles of resistant and susceptible mice (Figure 1 b); the ANOSIM R value between *0401 (n = 22) and *0402 (n = 21) males was only 0.145 (P<0.001). In contrast, differences in fecal microbiome structure were more evident between *0401 (n = 19) and *0402 (n = 24) females (ANOSIM R statistic = 0.436, P<0.001), (Figure 1 c and d). DRB1*0402 mice showed dynamically different fecal microbiomes based on sex (males and females, n = 21 and 24 respectively and age (<4 and >4 months old, n = 27 and n = 18 respectively) factors (ANOSIM R-statistic = 0.302 and 0.423 for sex and age differences respectively, P<0.001) (Figure 2a and 2b). Unlike arthritis-resistant *0402 mice however, the structure of the fecal microbiomes of *0401 mice lost these sex (males and females, n = 22 and n = 19 respectively) and age (<4 and >4 months old, n = 30 and n = 11 respectively) -driven differences (ANOSIM R values of 0.052 and 0.043 for gender and age differences respectively, P>0.1) (Figures 3 c and d).

### Specific gut commensals contribute to strain, sex and age differences in mice fecal microbiomes

A percentages-species contribution analysis (SIMPER) and Taxonomic search using RDP and NCBI databases allowed us to identify and characterize the relative abundance distributions of the five Operational Taxonomic Units (OTUs) that contributed more than 2.5% to the observed differences of the fecal microbiomes between resistant and susceptible transgenic mice (Table 1, Figure 3a). The phyla distributions of OTUs followed similar patterns when taking into account all of the 5,267 OTUs detected (Figure 3 b); at this taxonomic level, *0401 mice presented a more even Bacteroidetes/Firmicutes ratio (∼1∶1) than *0402 mice (∼2∶1).

**Table 1:** Identity of the main OTUS's driving strain, age and gender differences among mice fecal microbiomes.

Phylogenetic Affiliation	Closest match in the RDP environmental sequence database and refseq_rna	% ID	Closest match in the nr/nt NCBI database	% ID	Closest uncultured match in the nr/nt NCBI database	% ID	No. of times detected in the data set
Actinobacteria	*Bifidobacterium pseudolongum* subsp. *globosum* strain JCM 5820	99	*Bifidobacterium pseudolongum* subsp. *globosum* strain 02-2	100	Uncultured Bifidobacterium sp. clone PP187-b15	100	36,261
	NR_043441.1		AY166515.1		GU902754.1		
Bacteroidetes	*Barnesiella intestinihominis* YIT 11860 strain YIT	84	Gram-negative bacterium cL10-2b-4	88	Uncultured bacterium clone RMAM0391	99	30,891
	NR_041668.1		AY239469.1		HQ319321.1		
Bacteroidetes	*Barnesiella viscericola* DSM 18177 strain JCM 13660	82	Gram-negative bacterium cL10-2b-4	87	Uncultured bacterium gene	95	35,585
	NR_041508.1		AY239469.1		AB626927.1		
Bacteroidetes	*Parabacteroides distasonis* strain JCM 5825	83	Gram-negative bacterium cTPY-13	89	Uncultured bacterium clone 16saw38-2d06.q1k	99	35,234
	NR_041342.1		AY239461.1		EF604593.1		
Firmicutes	*Allobaculum stercoricanis* DSM 13633	84	Bacterium HBND	87	Uncultured bacterium clone R-9085	89	34,042
	NR_042110.1		AY498748.1		FJ881077.1		

Phylotype identity was assigned using the RDP (Ribosomal Database Project) classifier at 80% Bayesian bootstrap cutoff from comparisons to the environmental survey sequence database. RNA entries from NCBI's Reference Sequence project (refseq_rna) and no longer “non-redundant” nucleotide collection of the NCBI were also used to assign phylotype.

**Figure 4:**
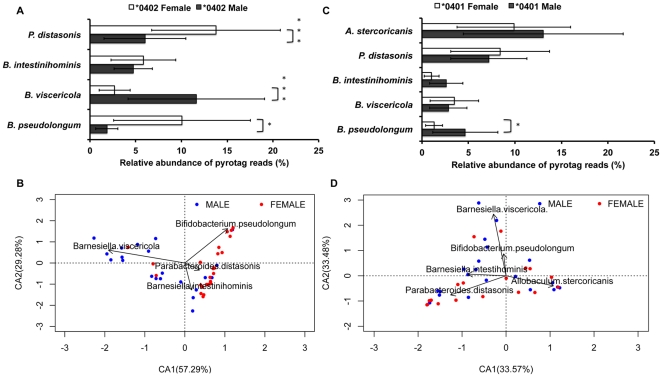
Sex based relative abundance of OTUs in the fecal microbiomes of *0401 and *0402 mice. (**a**) *0402 females (n = 24) show significantly higher relative abundances of *Bifidobacterium*-*Parabacteroides* OTUs compared to males (n = 21), whose microbiomes present significantly higher levels of *Barnesiella viscericola*. (**b**) Correspondence analysis plot displays sex-based correlation between OTUs in *0402 mice. (**c**) Significantly higher relative abundances of *Bifidobacterium sp*. were observed in *0401 males (n = 22) compared to females (n = 19), (**d**) despite loss of dynamic sex based differences in the fecal microbiomes of *0401 mice. Percentages in parentheses in the CA plots describe the amount of variation explained by each axis. Data are presented as mean ± S.E. *P<0.05, **P<0.01, ***P<0.001.

An OTU related to *Allobaculum* sp. (84% identity to *A. stercoricanis*) or an unclassified member of the Clostridiales (87% identity) was more abundant in disease-susceptible (*0401) mice compared to *0402 mice (P<0.00001). On the other hand, OTUs related to *Bifidobacterium*, *Barnesiella* and *Parabacteroides* spp., were more abundant in disease-resistant mice (P = 0.0029) (Figure 3 a). Data on the relative abundance of the OTU's driving microbiome differences between the two strains were used to construct a simple correspondence analysis plot (CA) showing the level of correlation between each OTU and members of either strain (Figure 4 c). Dimension 1 (Axis CA1) of the CA plot explained 42.94% of the total variation in the data and distinguished between susceptible mice, more correlated to the abundance of *Allobaculum* sp. and resistant mice, associated to greater proportions of the *Bifidobacteria* and the *Parabacteroides*-*Barnesiella* group.

Sex based differences in the fecal microbiomes of *0402 mice were driven mainly by *Bifidobacterium pseudolongum* subsp. *Globosum* and *Parabacteroides distasonis*, each being more prevalent in females (P = 0.018. and 0.00017 for *Bifidobacterium* and *Parabacteroides* respectively) (Figure 4a) while, *Barnesiella viscericola* was more abundant in males (P = 0.00018. n = 21 and 24 for resistant male and female mice respectively). Dimension 1 of a CA plot describing the level of association between either sex and specific OTUs explained 57.29% of the total variation in the data and showed high correlation between the relative abundance of *Barnesiella viscericola* and *0402 males, and of *Bifidobacterium pseudolongum and Parabacteroides distasonis* and *0402 females (Figure 4 b). Even though fecal microbiome of *0401 mice were dysbiotic and less dynamic (Figure 4 d), susceptible males did show significantly higher abundances of *B. pseudolongum* than susceptible females (P = 0.02, n = 22 and 19 for *0401 males and females respectively) (Figure 4 c). Age dependent fecal microbiota differences in resistant mice were driven mainly by *B. viscericola* that more abundant in older mice (<4 compared to >4 months, n = 18 and 27 respectively, P = 0.0001). However, the relative abundances of *Bifidobacterium* and *Parabacteroides* were not significantly different between older and younger *0402 mice (P = 0.766 and P = 0.0567 for *Bifidobacterium* and *Parabacteroides* respectively. There were no significant age-driven differences in the relative abundance of specific OTUs between susceptible mice.

### Arthritis susceptible DRB1*0401 mice show altered mucosal immune function and increased gut permeability compared to resistant DRB1*0402 mice

**Figure 5:**
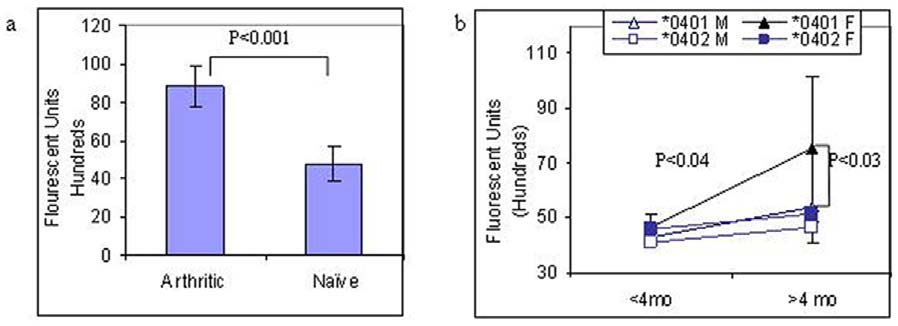
Gut permeability. (**a**) Transgenic arthritic mice showed significantly higher gut permeability compared to naïve mice (n = 5 each group). (**b**) Intestinal permeability in naïve *0401 and *0402 transgenic male and female mice at >4 and <4 months of age. *0401F<4 mo vs >4 mo, P<0.04; *0401F vs *0402F >4 mo, P<0.03 (n = 5–8 in groups).

We tested the hypothesis that dysbiosis in gut flora of *0401 mice may be associated with an altered intestinal permeability as well as a distinct expression of pro and anti-inflammatory cytokines in the gut as compared to arthritis-resistant mice, and that this dysbiosis may play a role in the pathogenesis of arthritis. A comparison of gut permeability between arthritic and naïve *0401 mice showed a significant increase in gut permeability in arthritic (n = 5) mice compared to naïve (n = 5) mice (P<0.0001, Figure 5a). To determine if the host genotype and gut flora may determine gut permeability, naïve male and female *0401 and *0402 mice were kept on a similar diet, cage bedding and room. Our data showed that there is a basal level of intestinal permeability which is significantly higher in *0401 mice as compared to *0402 mice and that it is age and sex-dependent in susceptible mice (Figure 5b). There was no difference in gut permeability between sexes at a young age (<4 months); however, as the *0401 mice aged, females (>4 months age) showed an increase in gut permeability as compared to the younger group, P<0.04 and older *0402 females (P<0.03). Resistant mice did not show any significant changes in gut permeability with age or sex (n = 5–8 in each group).

**Figure 6:**
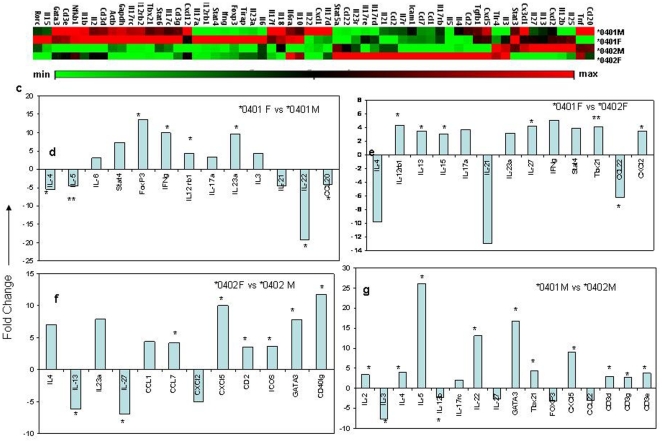
A)) Heat map showing expression levels of cytokines and chemokine transcripts in jejenum of *0401 and *0402 male and female mice (n = 3 in each group). (b) Comparison of fold change in gene transcript levels between *0401 females and males, (c) *0402 females and males, (**d**) females of each genotype and (e) males of each genotype. Results are given as fold-changes of mean copy-numbers relative to the mean copy-numbers of the comparative group. *P<0.05 and **P<0.01 and more. Data points with 3 or more fold differences and significance of more than P<0.05 are shown.

To determine if gut microbial composition is also associated with a different gut immune profile, we tested the jejuna of naïve mice for expression of cytokine and chemokine transcripts involved in the Th17 regulatory network by rtPCR (Figure 6 a–e, Figure S1, File S1). Susceptible *0401 females showed a distinct cytokine and chemokine profile as compared to males that was characterized with a significant increase in IL-23α and IFNγ along with a decrease in the regulatory cytokines IL-4, IL-22 and CCL20. Similarly, *0401 females showed more than 3 fold increased gene transcripts for Th17 cytokines IL-17, IL-23, IL-6 and Th1 cytokines IFNγ, Stat 4 and TBX21 while *0402 females had several fold increase in genes regulating Th2 cytokines and regulatory networks like ICOS, GATA3 and IL-4. *0401 male mice did not show an increase in transcripts for TH17 encoding genes compared to *0402 mice.

Next we determined if the relative abundance of the OTUs showing sex, age and strain differences in the transgenic mice were associated with specific cytokine/chemokine transcripts in jejuna (n = 12, 3 mice from each group, *0401 and *0402 males and females). Spearman correlation tests showed that *Bifidobacterium* species were negatively correlated to IL-17a (P = 0.06) and TBx21 (P = 0.004) transcript levels, while *Parabacteroides* species were negatively correlated to TBx21 (P = 0.012) (Table S1). The relative abundance of *Allobaculum* species were negatively correlated to CCL22 (P = 0.017) and IL-21 (P = 0.06). Since cytokines transcripts were studied in only six mice/strain, these results need to be interpreted with caution.

## Discussion

Interaction between genetic and environmental factors is required for predisposition to develop RA. The presence of bacterial DNA of gut-residing commensals in synovial fluid [3] has led to the hypothesis that certain mucosal bacteria may have a role in the susceptibility to develop arthritis. By taking advantage of transgenic mice that express the RA-susceptible *0401 transgene or the RA-resistant *0402 transgene, we have shown that genetic factors, along with sex background and disruption of gut microbiome may influence susceptibility and/or resistance toward developing arthritis in humanized mice. Our data on gut microbiome in genetically resistant mice is consistent with previous reports of age and sex based differences in the fecal microbiomes of healthy individuals [23], [24]. Resistant transgenic female mice, whose microbiomes were more similar, drove differences in microbiome structure between the two strains. This suggests that host genotype, rather than sex background, is a major regulator of gut microbial composition, an observation consistent with a recent report in inbred lines of mice [25]. However, as different human and murine models have shown, it is under debate whether the maintenance of adaptive immune mechanisms are mainly applied top-bottom (host-controlled) or bottom-up (driven by the gut microbiome), with gut bacterial communities acting as puppets or masters of the immune system [4], [26], [27], [28]. In this respect, the findings presented herein imply that the selection of a different T cell repertoire by two distinct HLA transgenes [29] modulates gut bacterial communities. This is consistent with studies that show increases in the incidence of autoimmune disorders driven by genotype, in such studies, interactions with specific commensals, harmless under immunocompetent conditions, could trigger disease [30], [31]. Conversely, studies with germ-free and specific pathogen-free mice have shown that disruptions to gut bacteria can promote increased levels of pro-inflammatory cytokine and interlukin-17 producing Th-17 cells, even in tissues distant to the gut [27], [31], [32], suggesting that global adaptive immune responses are also controlled by gut bacteria. Our data showed a bias towards TH1/TH17 cytokine expression with significant decrease in cytokine gene transcripts required for negative regulation of Th17 profile, like IL-4, IL-21 and IL-22, in *0401 females as compared to *0401 males and *0402 females. Interestingly CCL20 and CCL22 which are required for the generation of regulatory CD4 T cells and DCs [33], [34], [35], are reduced several fold in *0401 females as compared to *0401 males and *0402 females. A recent study showed that decreased levels of CCL20 during aging, are associated with isolated lymphoid dysfunction and mucosal immunosenescence [35]. These studies along with the present results that show a decreased CCL20 and loss of age differences in gut microbiome of *0401 females may confer mucosal dysfunction and immunosenescence. Arthritis-susceptible males however, showed a significant increase in Th1/Th2 but not in Th17 cytokines compared to resistant males, along with significant increases in genes for γδ T cells, suggesting a role for these cells in Th1/Th2 profile. Differences in chemokine gene transcripts observed between *0401 and *0402 mice and their correlation with microbiome profiles further supported our contention of dysbiosis leading to altered mucosal immune function in susceptible mice. However, these events could be contributing to pathogenesis independtly or in combination.

The significantly higher evenness index and even Bacteroidetes/Firmicutes ratios in *0401 as compared to *0402 mice, and their specific associations with either genotype, may be a reflection of an apparent dysbiosis phenomenon similar to that observed in other disease conditions [36]. Our observations suggest that *0402 mice maintain a homeostatic gut bacterial environment characterized by the overrepresentation and/or absence of specific microbiome structure. Specifically, members of Bacteroidetes and Actinobacteria occur twice as often as Firmicutes in *0402 mice as opposed to the stable ratios observed in *0401 mice. This observation implies that host genotype and environmental stimuli can cause expansion and/or contraction of certain members of a core or signature gut microbiome to modulate immunity. In the present study, microbiome differences between arthritis-susceptible and resistant mice are higher relative abundance of *Bifidobacterium pseudolongum* and members of the *Porphyromonadaceae* family in the latter that are positively correlated with regulatory cytokines. These observations support the importance of these taxa in the maintenance of a homeostatic gut microbiome. *Bifidobacteria* sp. have been recognized for their probiotic and immuno-modulating properties including down-regulating the expression of inflammatory pathways and enhancing gut barrier function [4], [36], [37], [38]. *Parabacteroides distasonis*, a commensal detected in higher proportions in arthritis-resistant mice, has been reported to reduce intestinal inflammation in murine models upon oral administration of its antigens [39] and is also involved in “educating” the immune system towards the tolerance of commensal antigens by enhancing Treg cell recognition mechanisms [40]. Thus in *0401 mice, particularly in females, reduced relative abundance of *Bifidobacterium sp.* and commensals from the *Porphyromonadaceae* family may lead to dysbiosis, enhanced pro-inflammatory responses, and a subsequent skewed immune response. Interestingly, the proportions of *Bifidobacterium* are inversely proportional or negatively correlated to the presence of *Allobaculum* sp. (Clostridiales order). Segmented filamentous bacteria (SFB), also from the Clostridiales, have been linked to immunosuppression and an increase of pro-inflammatory responses in arthritis driven by Th-17 cell proliferation [41], [42]. Although SFB were not detected in this study, the gut microbiomes of *0401 mice were characterized by a 7 fold increase in the relative abundance of *Allobaculum* sp. compared to *0402 mice. Based on 16S rRNA gene phylogenetic analysis, *A. stercoricanis* forms a branch closer to the XVI Clostridial cluster constituted by *Clostriudium innocum*, *Streptococcus pleomorphus* and several *Eubacterium* spp. [43]. In fact *C. innocum* was the closest hit in RDP database (80% ID), which could imply high phylogenetic concordance of our sequence to members of this Clostridiales group. Consistent with our results, *Clostridium* spp. have been reported to be enriched in immune-compromised subjects [44] and reductions in *C. innocuum* levels are reported upon oral administration of *Bifidobacterium* spp. [45]. A broader search in the non-redundant nucleotide NCBI database, also related this Clostridium-like sequence to a bacterium isolated from mice deficient in secretory antibodies (87% identity), in which there was increased recognition of gastrointestinal tract flora antigens by systemic antibodies and increased bacterial translocation [46]. Thus, these findings may indicate an association between inflammation and the high abundance of this OTU in *0401 mice.

Our data in humanized mice is also supported by a study in early RA patients, in which lower levels of Bifidobacteria and bacteria of the Bacteroides-Porphyromonas-Prevotella group were observed in RA patients compared to non-arthritic patients [7]. Members of the *Porphyromonadaceae* family are common dwellers of intestinal, oral and urogenital human and murine flora and have been identified as opportunistic commensals potentially pathogenic after immune disruption [47]. Herein, the relative abundance of members of the *Porphyromonadaceae* family (*Barnesiella and Parabacteroides* spp.) were significantly reduced in susceptible mice whenever the Clostridia-like bacterium became abundant. This observation implies that the Clostridia-like bacterium may induce disruption of normal commensals that are non-pathogenic under immunocompetent conditions. An increase in the gut permeability has been suggested to play a role in pathogenesis of arthritis [48]. Thus, the differences in microbiome and gut permeability seen in the *0401 and *0402 raises the possibility that, under certain conditions, disease causing bacteria like Clostridia, and other pathobionts or symbionts could produce translocation and a systemic immune response resulting in arthritis in those with a genetic susceptibility.

**Figure 7:**
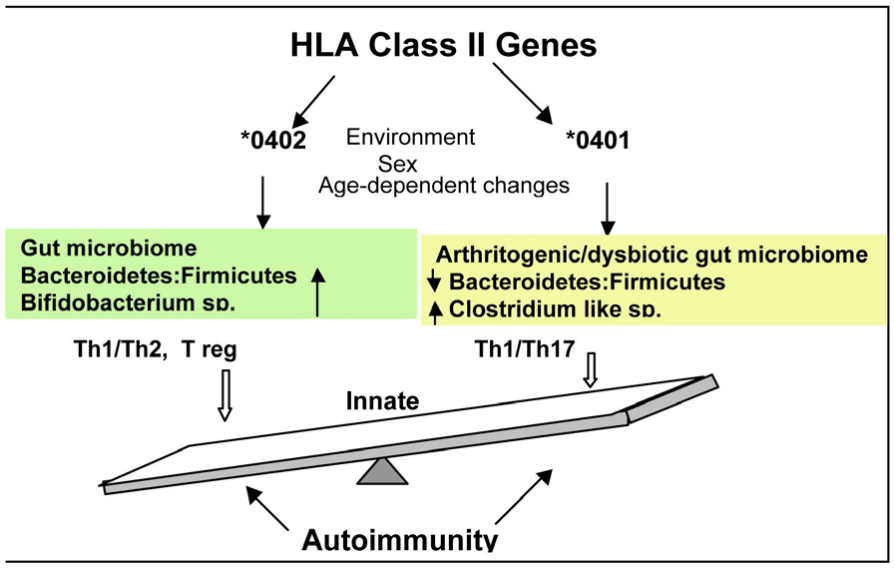
Role of the gut microbiome in susceptibility to arthritis. HLA genotype may shape the gut microbiome in an individual. The DRB1*0401 gene associated with predisposition to Rheumatoid arthritis, may induce a lower gut Bacteroidetes: Firmicutes ratio compared to that shaped by the DRB1*0402 gene, known to be associated with resistance to arthritis. This model suggests that a dysbiotic or arthritogenic gut microbiome may be dominated by a Clostridia-like bacterium (Firmicutes phylum) in susceptible individuals, while competent/tolerant immune responses are enhanced by increased abundances of *Bifidobacterium* spp. in resistance to RA. The gut microbiome has a crucial influence on maintaining homeostasis of the gut immune system by predicting pro-inflammatory (TH1/Th17) or anti-inflammatory (TH1/TH2) responses. Environmental triggers like smoking, diet and infectious agents along with sex hormones and age-dependent changes, may further modulate the gut immune system and enhance pro-inflammatory conditions in genetically susceptible individuals. In synthesis, an overall/systemic immune response generated by innate immune cells, may be originated at gut level and this response may be regulated by the gut microbiome via HLA genotype. This chain of events may determine the onset of autoimmune diseases like rheumatoid arthritis.

An interesting observation is the dynamic different microbiome structures based on age and/or sex background, driven by specific bacterial groups in *0402 mice. In contrast, this kind of microbial axis dynamism is completely lost in susceptible mice. This suggests the execution of specific, age/sex-based, immune regulation mechanisms in resistant mice. In this case, only females may have benefited from relative high abundance of *Bifidobacteria and Parabacteroides*. However, both *0402 males and females, regardless of age, seem to benefit from an absence of the Clostridia-like bacterium. This observation, along with the fact that in *0401 mice males may be taking advantage of significantly higher abundance of Bifidobacteria compared to females, raises the hypothesis that it is the loss of bacterial dynamism that is associated with disease susceptibility, particularly in females. A similar situation can be envisaged in human models in which beneficial microbiome may act as a modulator of proper immune response in the absence of host-genetic immune-regulators, and presence of pro-inflammatory bacteria potentially triggering disease. Although, specific molecular mechanisms remain largely unexplored, together, these results suggest that susceptibility to RA could be triggered by gut dysbiosis in genetically susceptible individuals. In turn, the onset of resistance may be characterized by a more dynamic microbiome, whose members expand and/or contract to provide sex and/or age based competent immune function, especially, in individuals that are not prone to develop RA. The hypotheses proposed herein (Figure 7), could be tested in future studies through experimentation with germ-free and SPF mice using various ways to manipulate gut microbiome and measure its impact in triggering disease. These approaches may include administration of probiotics which have been shown to alter intestinal microbiota and immune response and suppress CIA [49], [50]. Additionally, this model points a way forward to further probe gut microbial communities in various disease conditions, which would allow us to identify novel biomarkers and develop preclinical models to manipulate them for therapeutics.

## Materials and Methods

### Transgenic mice

The generation of DRB1*0401and DRB1*0402 transgenic (Tg) mice has been described previously [20], [29]. Aβ°.DRB1*0401 and Aβ°.DRB1*0402 mice were mated with MHCII^Δ/Δ^ (AE°) mice [51] to generate AE°.DRB1*0401 and AE°.DRB1*0402 mice. Mice of both sexes (8–12 weeks of age) used in this study were bred and maintained in the pathogen-free Immunogenetics Mouse Colony at the Mayo Clinic, Rochester, MN in accordance with the Institutional Animal care and use Committee (IACUC). For convenience, DRB1*0402 mice will be referred to as *0402, and DRB1*0401 mice as *0401.

The expression of DR on PBLs of transgenic mice was analyzed by flow cytometry using mAbs L227 (anti-DR) conjugated antibodies to characterize transgene positive mice.

### 16 s rDNA analysis of Mice Fecal microbiome

Microbial DNA was extracted using the MoBio UltraClean Soil Kit (Mo Bio Laboratories Inc., Carlsbad, CA, USA) with a bead-beating step from fecal material of a single mouse. The V1–V3 region of the 16S ribosomal RNA gene was amplified by Polymerase chain reaction (22 cycles of 94°C (30 s), 48°C (30 s), 72°C (2 min)) using primers 27f (CGTATCGCCTCCCTCGCGCCATCAG-AGAGTTTGATYMTGGCTCAG; corresponding to nucleotides 8–27 of the Escherichia coli 16 s rRNA gene) and 534r (CTATGCGCCTTGCCAGCCCGCTCAG-[MID tag 1–15]-ATTACCGCGGCTGCTGGCA; corresponding to nucleotides 514–534 of the E. coli 16 s rRNA gene). The amplicons were subjected to pyrosequencing using 454 FLX-Titanium technologies at the UIUC KECK. The resulting sequences were processed using a combination of tools from Mothur [52] and custom Perl scripts. Preliminary quality control steps included the removal of sequences shorter than 400 nt with homopolymers longer than 6 nucleotides and all reads containing ambiguous base calls or incorrect primer sequences. Sequences were aligned against the silva database and then trimmed so subsequent analyses were constrained to the same portion of the 16S rDNA. Potentially chimeric sequences were detected using chimera slayer (http://www.mothur.org/wiki/chimera.slayer/) and removed. The remaining reads were pre-clustered to remove sequences that are likely to have derived from sequencing errors (http://www.mothur.org/wiki/Pre.cluster) and then clustered using Mothur's average algorithm. Taxonomic classification of each OTU (clustered at 97% sequence similarity) was obtained by Blastn alignments to NCBI RNA reference sequence and non-redundant nucleotide databases and with the Ribosomal Database project (RDP) multiclassifier at 80% Bayesian bootstrap cutoff from comparisons to the environmental survey sequence database. All new data were deposited in the sequence read archive (http://www.ncbi.nlm.nih.gov/sra/), accession number SRA043819. NMDS plots and SIMPER analyses were constructed based on Bray-Curtis distance metrics using Primer-E from normalized OTU-abundance data. Correspondence analysis was plotted using the ca package and bipartite network analysis from the R project statistical software [53].

### Induction and evaluation of CIA

To induce CIA, 8–12 weeks old *0401 transgenic mice and negative littermates were immunized with 100 µg of type II collagen (CII) (Chondrex Inc.) emulsified 1∶1 with complete Freunds' adjuvant H37Ra (CFA, Difco Laboratories, Detroit, MI) intradermally at the base of the tail as previously described for CIA protocol [18]. Mice were monitored for the onset and progression of CIA from 3–12 weeks postimmunization. The arthritic severity of mice was evaluated as described previously with a grading system for each paw from 0–3 as described [20]. Mice with a score of 2 or more were used as arthritic mice.

### Intestinal permeability

As gut permeability may be diet dependent, all transgenic mice were kept on standard diet. Changes in intestinal permeability were determined using 4-KDa FITC-labeled dextran. Mice were deprived of food for 3 hours, then gavaged with FITC–labeled dextran (0.6 mg/g body weight). Three hours later, mice were bled and serum collected. FITC-dextran content of the sera was determined by using a microplate reader with an excitation of 490 nm and emission detection at 525 nm. Gut permeability was tested in age and sex matched arthritic (7 weeks post-immunization with CII) and naïve (non-immunized) mice.

### RNA isolation and Real- time Polymerase chain reaction

Jejenum of naïve transgenic mice were isolated from 4 months old mice. After a midline celiotomy, the intestine was flushed with cold (4°C) phosphate buffered saline (PBS) to remove intraluminal content and jejunal segments were placed in RNA stabilization buffer (Qiagen). Total RNA from the isolated tissue was extracted using the RNeasy kit and protocol (Qiagen). cDNA was prepared using RT2 First Strand Kit cDNA Synthesis Kit and Primer Mixes (SABiosciences). The quantification of gene expression related to the Th17 Regulatory Network was performed using the RT^2^ Profiler PCR Array PAMM-0773 (SABiosciences) and the HT7900 Fast Real-Time PCR System (ABI). Product amplification was measured and analyzed according to the manufacturer's instructions.

### Statistical analyses

NMMDS (Non-metric Multi-dimensional scale) plots, SIMPER (Percentages-species contribution) analyses and ANOSIM (Analysis of similarities) were constructed based on Bray-Curtis distance metrics using Primer-E (PRIMER 5, version 5.2.7 (Primer-E Ltd., Plymouth, United Kingdom. Clark, 2005) from square root transformed OTU-abundance data. The ANOSIM procedure generates a test statistic, R, calculated as: R = (*r_B_−r_W_*)/[1/4*n*(*n*−1)], where n is the total number of samples, *r_B_* is the average of rank similarities arising from all pairs of replicates between different mice fecal samples groups (Strain, sex or age), and *r_W_* is defined as the average of all rank similarities among replicates within mice fecal samples groups. An R value of 1 indicates complete dissimilarity between groups; an R of 0 indicates a high degree of community similarity among groups.

Relative abundance of each OTU calculated as number of reads of a taxon/total number of reads in a sample was used to construct correspondence analysis plots using the ca package (50) from the R project statistical software. To assess significant differences in relative abundances of specific OTUs between strain, sex or age groups non-parametric Mann-Whitney *U*/Wilcoxon rank sum tests were conducted using the R project statistical tool. All data for the non-parametric tests were checked for homogeneity of error variances using the Brown-Forsythe test. Statistical significance was set to P<0.05. Non-parametric Spearman correlation coefficients were determined using the PROC COR procedure from the SAS software platform (SAS version 9.1.3; SAS Institute, Cary NC), appropriate significance level, error correction and power for all tests were determined using the pwr package from the R project statistical software. Significance difference in expression of gene transcripts for Th17 cytokines and regulating genes were analyzed by online tool available from the manufacturer for the PAMM0733 array (SABiosciences) and is reported as significant fold change difference of p<0.05 between groups. Difference in gut permeability between ages and sex was calculated by student's T test with significance set at p<0.05.

## Supporting Information

Figure S1 Heat map showing expression levels of cytokines and chemokine transcripts in jejenum of *0401 and *0402 male and female mice.
(TIF)

Table S1 **Correlation coefficients between OUT's and cytokine/chemokine transcript levels.**
(DOCX)

File S1 Differences in Th17 regulatory network transcripts in *0401 and *0402 male and female mice.
(XML)
