# Supplementary figures and images for: Loss of Sex and Age Driven Differences in the Gut Microbiome Characterize Arthritis-Susceptible *0401 Mice but Not Arthritis-Resistant *0402 Mice
Source: PLoS One. 2012 Apr 24;7(4):e36095. doi: 10.1371/journal.pone.0036095 (PMC3338357; doi:10.1371/journal.pone.0036095)

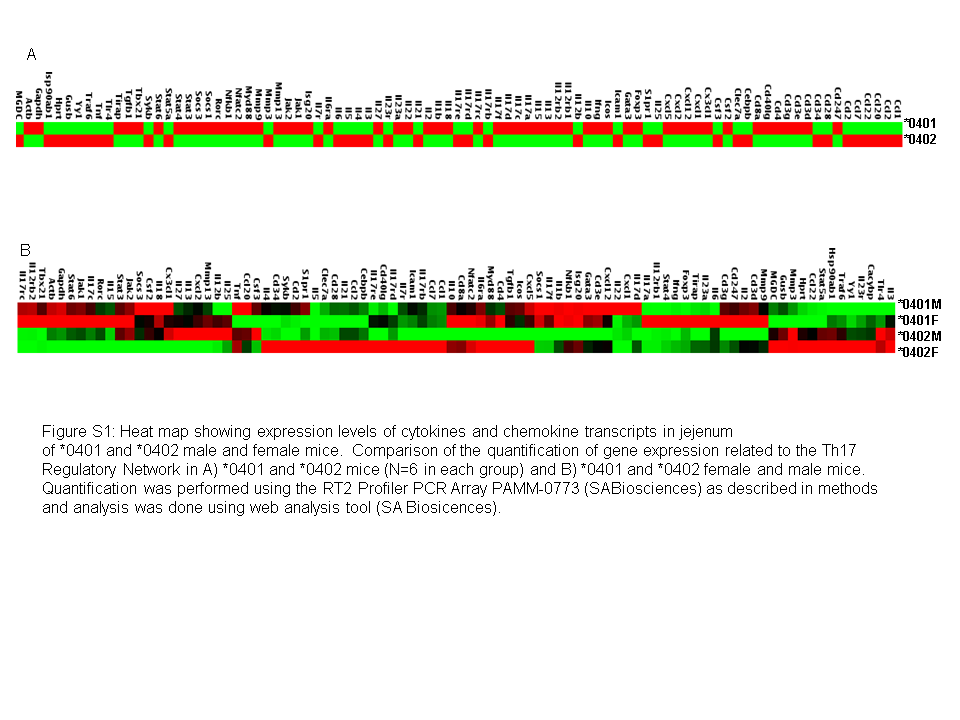

Supplement: Figure S1 — Heat map showing expression levels of cytokines and chemokine transcripts in jejenum of *0401 and *0402 male and female mice. (TIF) [file pone.0036095.s001.tif]
