# Supplementary material for: Loss of Sex and Age Driven Differences in the Gut Microbiome Characterize Arthritis-Susceptible *0401 Mice but Not Arthritis-Resistant *0402 Mice
Source: PLoS One. 2012 Apr 24;7(4):e36095. doi: 10.1371/journal.pone.0036095 (PMC3338357; doi:10.1371/journal.pone.0036095)
Supplement: Table S1 — Correlation coefficients between OUT's and cytokine/chemokine transcript levels. (DOCX) [file pone.0036095.s002.docx]

|  | **CCL20** | **CCL22** | **IL-17a** | **IL-23** | **IFNg** | **FoxP3** | **IL-13** | **IL-4** | **IL-21** | **IL-22** | **TBx21** | **ICOS** |
| --- | --- | --- | --- | --- | --- | --- | --- | --- | --- | --- | --- | --- |
| ***Bifidobacterium*** | -0.02098 | **0.727**** | **-0.552+** | -0.22 | -0.16 | -0.13 | -0.272 | 0.265 | **0.65*** | **-0.50+** | **-0.76**** | 0.02 |
| ***Barnesiella*** | -0.209 | -0.181 | 0.391 | -0.262 | 0.083 | -0146 | 0.02 | -0.20 | -0.48 | **-0.51+** | 0.104 | -0.475 |
| ***Parabacteroides*** | -0.143 | **0.755**** | -0.321 | -0.087 | -0.174 | -0.139 | -0.09 | 0.195 | **0.762**** | -0.356 | **-0.692*** | 0.0069 |
| ***Allobaculm*** | 0.153 | **-0.669*** | 0.284 | 0.265 | 0.22 | 0.156 | -0.266 | 0.128 | **-0.548+** | 0.366 | **0.768**** | 0.359 |

**Table S1 Correlation coefficients between OTUs and chemokine/cytokine transcript levels.**

Correlation coefficients were obtained based on the Spearman non-parametric approach (SAS version 9.1.3; SAS institute, Cary, NC). Six mice per strain were used for the analysis (3 males and 3 females) for a total of 12 individuals. Significant correlations are highlighted (**+P<0.1, *P<0.05, **P<0.01, ***P<0.001).**
